# DNA Damage and Inhibition of Akt Pathway in MCF-7 Cells and Ehrlich Tumor in Mice Treated with 1,4-Naphthoquinones in Combination with Ascorbate

**DOI:** 10.1155/2015/495305

**Published:** 2015-02-22

**Authors:** Fabiana Ourique, Maicon R. Kviecinski, Karina B. Felipe, João Francisco Gomes Correia, Mirelle S. Farias, Luiza S. E. P. W. Castro, Valdelúcia M. A. S. Grinevicius, Jaime Valderrama, David Rios, Julio Benites, Pedro Buc Calderon, Rozangela Curi Pedrosa

**Affiliations:** ^1^Laboratório de Bioquímica Experimental, Departamento de Bioquímica, Universidade Federal de Santa Catarina, Florianópolis, Brazil; ^2^Programa de Pós-Graduação em Ciências da Saúde da Universidade do Sul de Santa Catarina (UNISUL), SC, Brazil; ^3^Departamento de Ciencias Químicas y Farmacéuticas, Universidad Arturo Prat, 2120 Avenida Arturo Prat, Iquique, Chile; ^4^Toxicology and Cancer Biology Research Group, Louvain Drug Research Institute, Université Catholique de Louvain, 73 Avenue E. Mounier, GTOX 7309, 1200 Brussels, Belgium

## Abstract

The aim of this study was to enhance the understanding of the antitumor mechanism of 1,4-naphthoquinones and ascorbate. Juglone, phenylaminonaphthoquinone-7, and 9 (Q7/Q9) were evaluated for effects on CT-DNA and DNA of cancer cells. Evaluations in MCF-7 cells are DNA damage, ROS levels, viability, and proliferation. Proteins from MCF-7 lysates were immunoblotted for verifying PARP integrity, *γ*H2AX, and pAkt. Antitumor activity was measured in Ehrlich ascites carcinoma-bearing mice. The same markers of molecular toxicity were assessed *in vivo*. The naphthoquinones intercalate into CT-DNA and caused oxidative cleavage, which is increased in the presence of ascorbate. Treatments caused DNA damage and reduced viability and proliferation of MCF-7 cells. Effects were potentiated by ascorbate. No PARP cleavage was observed. Naphthoquinones, combined with ascorbate, caused phosphorylation of H2AX and inhibited pAkt. ROS were enhanced in MCF-7 cells, particularly by the juglone and Q7 plus ascorbate. Ehrlich carcinoma was inhibited by juglone, Q7, or Q9, but the potentiating effect of ascorbate was reproduced *in vivo* only in the cases of juglone and Q7, which caused up to 60% inhibition of tumor and the largest extension of survival. Juglone and Q7 plus ascorbate caused enhanced ROS and DNA damage and inhibited pAkt also in Ehrlich carcinoma cells.

## 1. Introduction

Current chemotherapy for cancer has limited efficacy and safety. It causes side effects, and the tumor cells often become resistant [1]. Studies dedicated to the development of novel agents for the treatment of cancer are highly encouraged. Quinoid compounds have been widely studied for potential cancer therapies [2–4]. It is known that some quinoid compounds can induce oxidative stress lethal to cancer cells. Some quinoid compounds can also bind to DNA due to their electrical charge. Doxorubicin is an example of that: it can increase the generation of reactive oxygen species (ROS) and bind to DNA [5].

DNA is the target for most anticancer drugs. In cancer cells, the interactions between such drugs and DNA result in cell damage, block cell division, and lead to cell death [6]. These molecules can bind DNA by externally interacting with the minor or major grooves. They can also act as intercalating agents by inserting between the base-pairs and thus reducing the helical twist and lengthening the DNA [7]. The interaction of molecules with DNA has been applied in therapeutic approaches that use the modulation of gene transcription and suppression of the replication to kill tumor cells [8].

Naphthoquinones have been a subject of study because of their use in a variety of medical and biological applications. As part of our ongoing studies concerning the preparation of potential biologically active compounds, 1,4-naphthoquinones, such as juglone (5-hydroxy-1,4-naphthoquinone), 2-(4-hydroxyaniline)-1,4-naphthoquinone (Q7), and 2-(4-methoxyaniline)-1,4-naphthoquinone (Q9) were screened because they possess anticancer potential* in vitro* [3, 4]. It has been shown that their anticancer effects can be potentiated by combining them with pharmacological doses of ascorbate. The use of ascorbate as an anticancer has been addressed by a large number of papers. Pharmacological ascorbate itself has been already proposed as a pro-drug for the delivery of H_2_O_2_ to tumors [9, 10]. But data provide support for investigating the use of pharmacological ascorbate as an adjuvant because it was shown that ascorbate triggers synergism with chemotherapy agents such as gemcitabine for instance [11].

The activity of formulations including quinoid compounds and ascorbate has been traditionally attributed to the enhanced generation of cellular ROS to levels that are above the protection capacity of cancer cells [12]. The current study enhances the understanding of the antitumor mechanism of 1,4-naphthoquinones and ascorbate. The data highlight the effects of these molecules on the DNA and shows that when juglone or Q7 are administered together with ascorbate, a very damaging formulation is directed against the DNA of cancer cells* in vitro* and* in vivo*. The DNA of cancer cells thus degrades due to an oxidative stress caused by molecules that bind and intercalate into its strands. As a result, cell proliferation and tumor growth are inhibited, as shown in the following* in vitro* and* in vivo.*

## 2. Materials and Methods

### 2.1. Chemicals and Antibodies

The 1,4-naphthoquinones Q7 and Q9 were synthesized by amination of 1,4-naphthoquinone with the respective arylamines, under aerobic conditions using CeCl_3_·7H_2_O as the Lewis acid catalyst, as previously described [13]. Dulbecco's modified Eagle medium (DMEM), fetal bovine serum (FBS), and antibiotics were purchased from Gibco (USA). The following stuff was purchased from Sigma-Aldrich: juglone, sodium ascorbate, calf thymus DNA (CT-DNA), agarose, dimethyl sulfoxide (DMSO), 2′,7′-dichlorofluorescein diacetate (DCFH-DA), 5,5′-dithio-bis(2-nitrobenzoic acid) (DTNB), bovine serum albumin (BSA), ethidium bromide (EtdBr), thiobarbituric acid, and the protease inhibitor cocktail. The phosphatase inhibitor cocktail was from Calbiochem (Merck Biosciences). Rabbit polyclonal antibodies against poly (ADP-ribose) polymerase (PARP), phosphorylated histone gamma H2AX (*γ*H2AX), and phosphorylated Akt (pAkt) were from Santa Cruz Biotechnology, Inc. (USA). Mouse *β*-actin antibody, the secondary antibodies, and the kit for chemiluminescence detection of horseradish peroxidase- (HRP-) coupled antibodies were from Millipore (USA).

### 2.2. Effects on CT-DNA* In Vitro*

DNA intercalation was examined by fluorescence measurements using a TECAN Infinity M200 microplate reader. CT-DNA (10 *μ*M) was saturated with ethidium bromide (3 *μ*M) in 50 mM phosphate buffer containing 0.1 M NaCl (pH 7.4). Fluorescence titrations were conducted by maintaining constant concentrations of CT-DNA and ethidium bromide and varying the concentrations of 1,4-naphthoquinones (0–40 *μ*M). The excitation/emission wavelengths were 492 nm and 620 nm, respectively [14].

Oxidative cleavage of CT-DNA was evaluated by the method proposed by Jun et al. [15] using 2-thiobarbituric acid. CT-DNA (0.5 mM) in 50 mM phosphate buffer (pH 7.2) was exposed to 10 *μ*M of 1,4-naphthoquinones and ascorbate 1 mM and incubated at 37°C for 2 h. After incubation, 2-thiobarbituric acid solution (1%) in 50 mM NaOH and glacial acetic acid were added (1 : 1 : 1) and incubated at 100°C for 30 min. After cooling, the absorbance was measured at 532 nm. Blanks contained all components except 1,4-naphthoquinones and ascorbate. The control had free radical generators [Fe(EDTA)]^2−^ (100 *μ*M) and hydrogen peroxide (10 mM).

### 2.3. Effects on the DNA of MCF-7 Cells* In Vitro*

Human breast carcinoma MCF-7 cells were purchased from the Rio de Janeiro cell bank, Brazil. Cells were cultured at 37°C under 5% CO_2_ atmosphere with 95% air humidity. DMEM was used supplemented with 10% FBS, penicillin (100 U/mL), and streptomycin (100 *μ*g/mL). The effects on the DNA of MCF-7 cells were examined by the comet assay [16] and the phosphorylation on histone H2AX. The occurrence of phosphorylation on serine 139 of histone H2AX, namely, *γ*H2AX, has been widely used as a sensitive marker of double-strand DNA breaks [17]. Gamma-H2AX was measured in whole MCF-7 cell homogenates through immunoelectrophoresis using the method described in Section 2.5. PARP and pAkt were evaluated by the same method. MCF-7 cells were used primarily because they allow studying the effects of compounds on the proliferation and they express H2AX and Akt [18, 19].

### 2.4. The Comet Assay

Treated cells were suspended in 0.75% low-melting point agarose and then deposited on the surface of a slide containing a thin layer of 1.5% agarose and allowed to set for 10 min at room temperature. The slides were submerged for 2 h in a lysis solution (2.5 M NaCl, 10 mM Tris, 100 mM EDTA, 1% Triton X-100, 10% DMSO, and pH 10.0) and then subjected to horizontal electrophoresis at 300 mA, 8°C, for 20 min in a tank with buffer (300 mM NaOH, 1 mM EDTA, and pH 13). A neutralizing solution (0.4 M Tris-HCl, pH 7.5) was added (3 times), followed by washing in water and drying at 37°C. A fixing solution (15% trichloroacetic acid, 5% ZnSO_4_, and 5% glycerol) was then added for 10 min, followed by washing and drying. The slides were stained with ethidium bromide (0.5 mg/mL) and analyzed under a fluorescence microscope. Each nucleus received a fluorescence value in the 0–4 range (arbitrary units: 0—undamaged, 4—maximally damaged) [20].

### 2.5. Immunoblotting Assays

After treatment, cells were washed with phosphate buffered saline (PBS) and lysed in RIPA buffer (50 mM Tris-Cl, pH 7.4, 150 mM NaCl, 1% NP40, 0.25% Na-deoxycholate, and 1 mM phenylmethylsulfonyl fluoride) supplemented with 1% protease inhibitor and 3% phosphatase inhibitor cocktails. After denaturation in Laemmli buffer (60 mM Tris-Cl, pH 6.8, 2% sodium dodecyl sulfate (SDS), 10% glycerol, 5% *β*-mercaptoethanol, and 0.01% bromophenol blue), equal amounts of protein (30 *μ*g) from whole cellular homogenates were subjected to polyacrylamide gel electrophoresis (SDS-PAGE), followed by electroblotting to polyvinylidene fluoride (PVDF) membranes. After blocking, the membranes were incubated overnight with the primary antibodies. The membranes were washed and incubated with the secondary antibodies for 1 h. Immunodetection was performed using the enhanced chemiluminescence (ECL) detection kit (Millipore, USA) for HRP-coupled secondary antibodies. Beta-actin served as a loading control.

### 2.6. Effects on MCF-7 Cell Viability and Proliferation

Cytotoxicity was measured using the tetrazolium salt (MTT) assay [21]. Briefly, 10^4^ cells/well were plated onto 96-well plates. At confluence, the cells were exposed to juglone, Q7, and Q9 (0–80 *μ*M) in the absence or presence of ascorbate (1 mM) for up to 24 h. The cells were then washed twice with PBS and incubated for 2 h with MTT (0.5 mg/mL). The formazan crystals were solubilised by adding DMSO (100 *μ*L/well), and the colored solutions were read at 550 nm. Three independent experiments were conducted, and the results are presented as EC_50_ values.

The effects on cell proliferation were examined by the colony formation assay, according to Franken et al. [22]. Cells (500) were treated for 2 h with the compounds. They were then washed twice with warm PBS, and fresh medium was added. After 15 days, the cells were stained by crystal violet, and colonies with more than 50 cells were counted.

### 2.7. Levels of MCF-7 Intracellular ROS

Intracellular ROS were measured as reported by Glorieux et al. [23]. Cells (15.000) were loaded with 10 *μ*M DCFH-DA in Hank's balanced salt solution (HBSS) at 37°C and incubated for 30 min. Excess DCFH-DA was removed by washing with fresh HBSS. The cells were incubated for 2 h with the test compounds, washed twice with HBSS, and then 100 *μ*L of HBSS was added to each well. The fluorescence intensity was measured with a TECAN Infinity M200 microplate reader at 485 nm for excitation and 530 nm for emission.

### 2.8. Antitumor Activity* In Vivo*

Male BALB/c inbred mice (20–22 g) received water and food* ad libitum*. Procedures were conducted in accordance with legal requirements and with the approval of the local ethics committee (UFSC/PP00784). Previous tests were conducted to select safe doses of 1,4-naphthoquinones. Ascorbate was administered at doses 100 times higher. On day zero, Ehrlich carcinoma cells (5 × 10^6^) were inoculated into the abdomen of mice from nine groups (*n* = 12). Treatments were done via intraperitoneal injections every 24 h for 9 days. The control group received saline injections and the positive control group received doxorubicin (1.2 mg/kg). Test groups received juglone, Q7, or Q9 (1 mg/kg) and/or ascorbate (100 mg/kg). After treatment, the inhibition of tumor growth was measured, based on changes in the abdominal circumference [24]. The percentage of increased life span was calculated by recording mortality on a daily basis for 30 days, according to the method of Kaplan and Meier [25]. Samples of ascitic fluid were collected 2 h after the last dose and immediately processed. The molecular effects* in vivo* were assessed again at level of DNA through the comet assay and gamma-H2AX. As done* in vitro* with MCF-7 cells, also* in vivo* Akt-pathway was evaluated in Ehrlich tumor after treatments through electrophoresis and immunoblotting as described in Sections 2.4 and 2.5. Lipid peroxidation was estimated by measurement of malondialdehyde (MDA) formation using the thiobarbituric acid method [26].

### 2.9. Data Analysis

In general, the assays were performed in triplicate.* In vitro* assays were repeated at least three times. The results are presented as the means ± standard deviation or as percentages. The data were analyzed by the analysis of variance (ANOVA) test followed by the Bonferroni test. Comparisons were performed using the GraphPad Prism software (San Diego, USA). Values of *P* < 0.05 were considered statistically significant.

## 3. Results and Discussion

**Figure 1:**
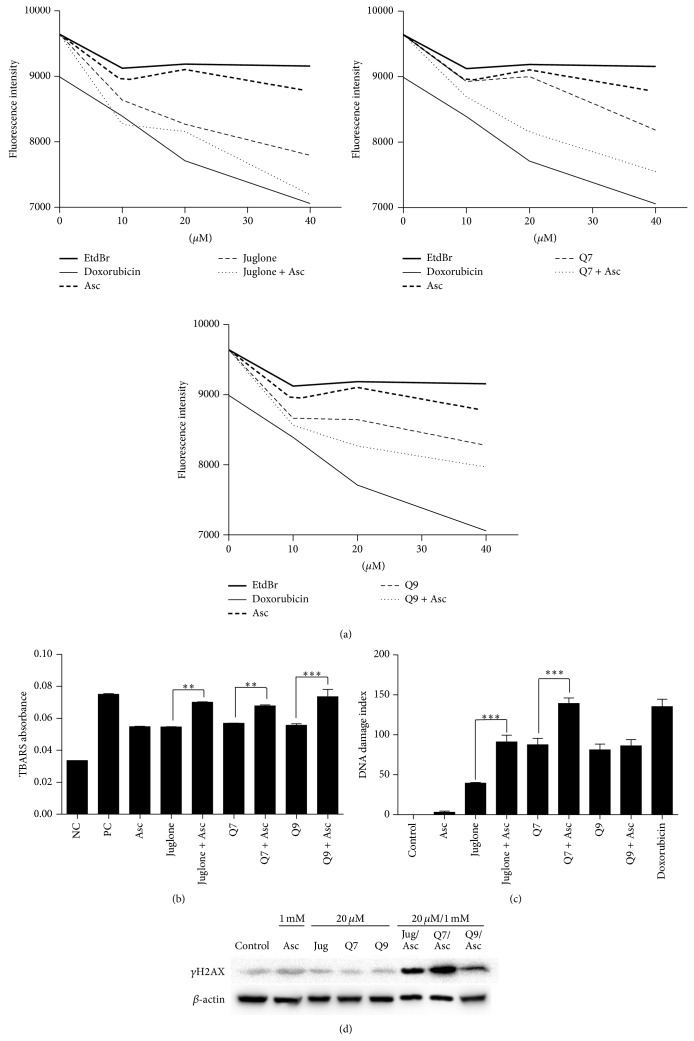
The fluorescence of CT-DNA stained with ethidium bromide is reduced by doxorubicin and juglone, Q7, or Q9, mainly in presence of ascorbate (Asc) (a). Absorbance of thiobarbituric reactive species (TBARS) in CT-DNA treated with juglone, Q7, or Q9 alone and combined with ascorbate. Negative control (NC): phosphate buffer. Positive control (PC): [Fe(EDTA)]^2−^/H_2_O_2_ (b). DNA damage index was determined by the comet assay in MCF-7 cells treated for 24 h with juglone 75 *μ*M, Q7 50 *μ*M, or Q9 50 *μ*M with or without ascorbate 1 mM (c). *γ*H2AX was assessed by immunoelectrophoresis in MCF-7 cells treated for 2 h with juglone (Jug), Q7, or Q9 at 20 *μ*M with or without ascorbate (Asc) at 1 mM (d). (∗∗) and (∗∗∗) denote statistical differences at *P* < 0.01 and *P* < 0.001 compared to indicated treatments.

Intercalation of juglone, Q7, and Q9 into CT-DNA was examined by using the fluorescent intercalating agent ethidium bromide. Compounds that are able to intercalate into DNA compete with ethidium bromide and reduce its fluorescence when read in a fluorimeter. As show in Figure 1(a), when DNA and ethidium bromide were incubated with juglone, Q7, or Q9, the fluorescence was reduced, indicating that the compounds can intercalate into CT-DNA. The intercalating capacity of juglone, Q7, and Q9 was always higher when the incubations were performed in the presence of ascorbate. As expected, doxorubicin, which is a known intercalating agent, also reduced the fluorescence signal [27].

CT-DNA treated with juglone, Q7, or Q9 underwent oxidative cleavage as shown by data in Figure 1(b). Free radicals can attack DNA at C4 of desoxyribose causing cleavage and generation of products of degradation such as base propenal. This can be detected because base propenal reacts with 2-thiobarbituric acid producing color [28]. Data in Figure 1(b) shows that the absorbance resulting from thiobarbituric acid species (TBARS) was increased in CT-DNA treated with the free radical generators [Fe(EDTA)]^2−^/H_2_O_2_ as well as it occurred in CT-DNA treated with juglone, Q7, or Q9. TBARS absorbance was always higher when CT-DNA was treated with the naphthoquinones combined with ascorbate (Figure 1(b)).

The effects on the DNA of MCF-7 cells were assessed by immunoelectrophoresis for *γ*H2AX and the comet assay. Gamma-H2AX is required for many proteins during the DNA damage and repair response [17].  Figure 1(d) shows that, individually, neither ascorbate, juglone, Q7, nor Q9 enhanced the phosphorylated protein band corresponding to *γ*H2AX. The level of *γ*H2AX was enhanced only when MCF-7 cells were treated with the compounds combined with ascorbate (1 mM). Consistent with these findings, the comet assay showed that juglone, Q7, and Q9 induced the fragmentation of MCF-7 DNA on their own, but the addition of ascorbate increased DNA fragmentation by approximately 2-fold in the case of juglone and Q7 (Figure 1(c)).

The effects of juglone, Q7, and Q9, with and without ascorbate, were then examined on viability and proliferation in MCF-7 cells. The MTT assay showed that ascorbate (1 mM) alone did not reduce the viability of the MCF-7 cells. The cytotoxicity of juglone, Q7, and Q9 was determined, showing EC_50_ values of 61, 41.6, and 50 *μ*M, respectively. The addition of ascorbate together with the naphthoquinones reduced the EC_50_ values by approximately 2-fold (to 28, 26.3, and 25.4 *μ*M for juglone, Q7, and Q9, resp.) and thus enhanced cytotoxicity (Table 1).

**Figure 2:**
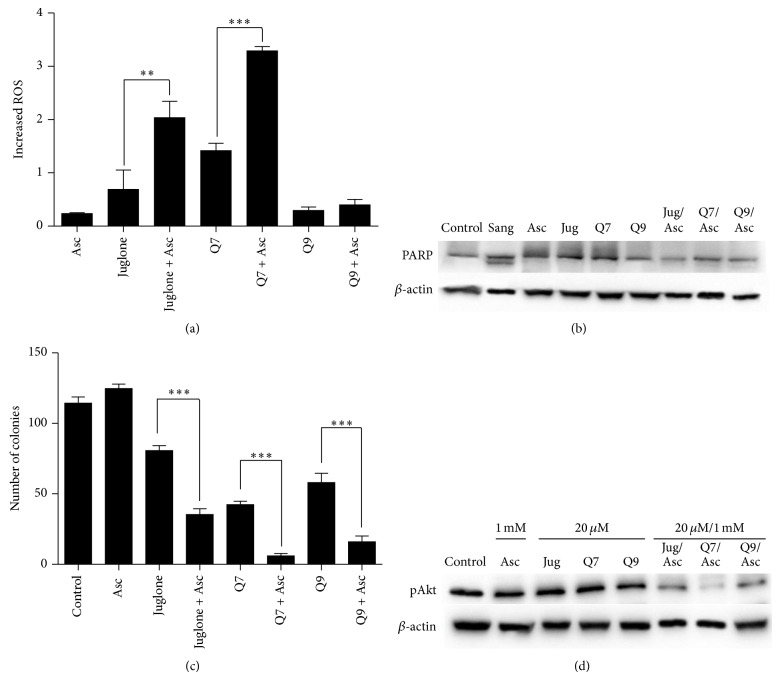
ROS were measured in MCF-7 cells treated for 2 h with juglone, Q7 or Q9 at 10 *μ*M with or without ascorbate 1 mM (a). Integrity of PARP protein in MCF-7 cells treated with juglone (Jug), Q7 or Q9 at 20 *μ*M with or without ascorbate (Asc) 1 mM for 24 h. Sanguinarine (Sang) 5 *μ*M was used as a positive control of apoptosis (b). Colony-forming units of MCF-7 cells treated with juglone, Q7 or Q9 at 10 *μ*M with/without ascorbate 1 mM for 2 h (c). Phosphorylated Akt (pAkt) was assessed by immunoelectrophoresis in MCF-7 cells treated for 24 h (d). Data were obtained from three independent experiments. (∗∗) and (∗∗∗) denote statistical differences at *P* < 0.01 and *P* < 0.001 compared to nontreated control cells or between indicated treatments, respectively.

**Figure 3:**
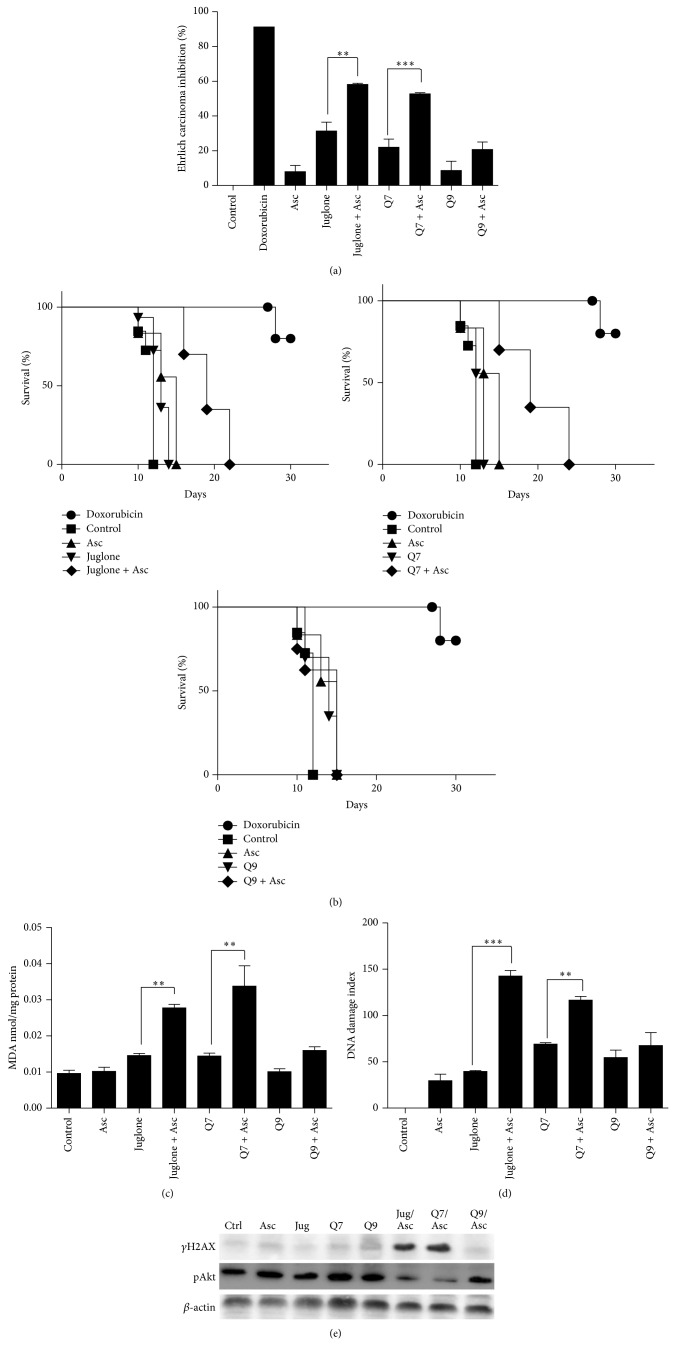
Ehrlich ascites carcinoma inhibition (a) and survival time (b) in animals treated with juglone, Q7, or Q9 at 1 mg/kg alone or in combination with ascorbate (Asc) at 100 mg/kg. Doxorubicin was administered at 1.2 mg/kg for the positive control. Lipid peroxidation (c), DNA damage (d), and phosphorylated proteins Akt (pAkt) and H2AX (*γ*H2AX) in ascitic cells from mice (e). (∗∗) and (∗∗∗) denote difference at *P* < 0.01 and *P* < 0.001 compared to the negative control or between indicated treatments.

Ascorbate activates the redox cycling of quinones generating semiquinone radicals and ROS. In T24 cells, it was previously shown that ascorbate (1 mM) administered with juglone, Q7, and Q9 caused the appearance of semiquinone and elevated levels of intracellular ROS [3, 4]. If a similar phenomenon occurs in MCF7 cells, DNA damage would result from not only binding and intercalation but also from an oxidative attack. Doxorubicin acts by such a mechanism and kills cancer cells through both DNA intercalation and ROS induction [29].  Figure 2(a) shows that the ROS levels in MCF-7 cells were elevated by the compounds, particularly by juglone and Q7. ROS generation induced by juglone and Q7 was enhanced approximately 3-fold in the presence of ascorbate. On the other hand, ascorbate had less impact in terms of ROS induction when administered together with Q9 on MCF-7 cells. Later, the formulations containing Q9 or Q9 plus ascorbate were proven as poor inducers of oxidative stress in both MCF-7 cells (Figure 2(a)) and Ehrlich ascites carcinoma in mice (Figure 3(c)). Ascorbate caused some increase in terms of oxidative damage by Q9 only when our treatments were done on CT-DNA, a noncellular system (Figure 1(b)).

Cell death was further studied based on PARP cleavage, as shown in Figure 2(b). Compounds that are able to induce apoptosis cleave the PARP protein, replacing the full-length 116 kD protein with a cleaved fragment of approximately 89 kD [30]. Sanguinarine (5 *μ*M), a flavonoid known to induce apoptosis, was used as a control [31]. PARP cleavage was observed only in sanguinarine-treated cells and not in cells treated with juglone, Q7, or Q9, with or without ascorbate (Figure 2(b)).

The effect on MCF-7 cell proliferation is shown in Figure 2(c). Juglone, Q7, and Q9 administered alone inhibited cell proliferation, as indicated by a reduction in the number of colony-forming units in comparison with nontreated cells. In all cases, the inhibition of proliferation was increased up to 3-fold when the naphthoquinones were administered in combination with ascorbate (1 mM) (Figure 2(c)). These data corroborate literature when considered that DNA intercalators can block cell division [6]. To examine whether the PI3K/Akt/mTOR signalling pathway was affected by the treatments in MCF-7 cells, the levels of the active phosphorylated form of Akt (pAkt) were determined (Figure 2(d)). The PI3K/Akt pathway regulates several biological processes, including cell survival, proliferation, and differentiation. Upregulation of this pathway is observed in several types of cancer, and it can be associated with uncontrolled cell proliferation [32–34]. As demonstrated in Figure 2(d), significant inhibition of the pAkt occurred in MCF-7 cells following treatment, most notably with juglone or Q7 in the presence of ascorbate.

Following these* in vitro* assays, some* in vivo* effects were studied in Ehrlich ascites carcinoma-bearing mice. Ehrlich ascites carcinoma was chosen to be used* in vivo* initially to verify whether some effects observed* in vitro* were reproducible* in vivo*. But, also to evaluate whether the effects were consistent only with MCF-7 cells* in vitro* or they could be repeated against a different tumor cell line.  Figure 3(a) presents the levels of tumor growth inhibition. Some inhibition on Ehrlich carcinoma was caused in animals treated with juglone, Q7, or Q9. But the formulations of juglone or Q7 plus ascorbate had the most potent activity and achieved up to 60% of inhibition of tumor growth, approaching the effect of doxorubicin, which caused up to 90% inhibition. Actually, considering data related to tumor growth and survival, it is possible to suggest that the potentiating effect of the combined treatment with ascorbate was reproduced* in vivo* with statistical difference only in the case of juglone and Q7. The ability to increase the duration of animal survival is one of the most reliable criteria for evaluating potential antitumor drugs [35].  Figure 3(b) presents graphs relating the number of days after tumor inoculation to the percentage of survivors following treatment; an increase in the area under the curves indicates an increase in survival. The smallest area in the graphs corresponds to the group of nontreated animals. In general, these animals started to die on day 10, and by day 12, no survivors remained. The survival time was extended by 2 to 6 days when the animals received treatments with individual compounds (juglone, Q7, or Q9). These animals generally died before day 20. The largest area under the curves corresponds to the treatments conducted with juglone or Q7 in combination with ascorbate (Figure 3(b)). The synergistic effect between these drugs occurred also* in vivo*.

Figures 3(c), 3(d), and 3(e) present data of molecular toxicity on Ehrlich tumor in mice. Figure 3(c) depicts levels of MDA; a biomarker of lipid peroxidation. This was the end-point measurement done to verify oxidative stress caused by the treatments* in vivo*. MDA levels were increased significantly by the combined treatments done with juglone or Q7 and ascorbate. Data in Figure 3(d) corroborate with data from Figure 3(c), because they show that DNA fragmentation was increased significantly* in vivo* only when animals received juglone or Q7 plus ascorbate. Finally, data shown in Figure 3(e) provide concise evidences which demonstrate that juglone and Q7 plus ascorbate can cause DNA damage and pAkt inhibition in Ehrlich tumor in mice. Thus, the effects are not restricted to MCF-7 cells* in vitro*.

**Figure 4:**
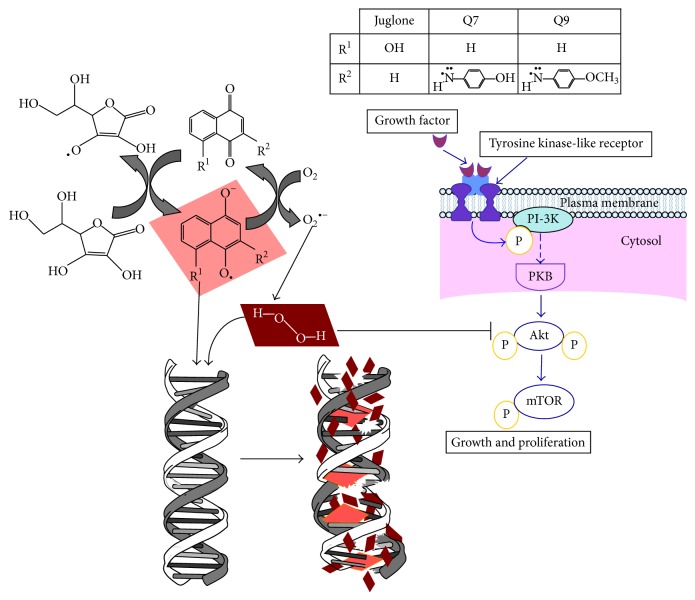
Antitumor actions of juglone, Q7, and Q9 administered in combination with ascorbate against MCF-7 cells and Ehrlich ascites carcinoma in mice. The effects are the result of intercalation and oxidative attack on DNA of tumor cells and inhibition of Akt pathway.

Data from this study are summarized illustratively in Figure 4. Although all 1,4-naphthoquinones presented some activity, primarily juglone and Q7 administered in combination with ascorbate trigger the generation of free radicals, the semiquinone form of the naphthoquinone and dehydroascorbate. The resulting semiquinone form intercalates into DNA. The radicals can attack DNA, which is degraded. In cancer cells, these events are accompanied by an inhibition on Akt pathway. The formulation of juglone or Q7 with ascorbate showed the most promising activity* in vivo*.

## 4. Conclusion

Overall, it is possible to conclude that primarily juglone and Q7 combined with ascorbate have significant antitumor effects against MCF-7 cells* in vitro* and Ehrlich-ascites carcinoma in mice. The effects are the result of intercalation and oxidative attack on DNA of tumor cells and inhibition of Akt pathway.

## Figures and Tables

**Table 1:** EC_50_ values obtained by the MTT assay. MCF-7 cells were treated for 24 h with juglone, Q7, or Q9 (5–80 *μ*M) with or without ascorbate 1 mM. Data from three independent experiments.

	EC_50_ (*μ*M)		
	Juglone	Q7	Q9
	61	41.6	50
*plus* ascorbate 1 mM	28	26.3	25.4
